# Global proteome analyses of phosphorylation and succinylation of barley root proteins in response to phosphate starvation and recovery

**DOI:** 10.3389/fpls.2022.917652

**Published:** 2022-08-18

**Authors:** Juncheng Wang, Chengdao Li, Lirong Yao, Zengke Ma, Panrong Ren, Erjing Si, Baochun Li, Yaxiong Meng, Xiaole Ma, Ke Yang, Xunwu Shang, Huajun Wang

**Affiliations:** ^1^State Key Lab of Aridland Crop Science/Gansu Key Lab of Crop Improvement and Germplasm Enhancement, Lanzhou, China; ^2^Department of Crop Genetics and Breeding, College of Agronomy, Gansu Agricultural University, Lanzhou, China; ^3^Western Barley Genetics Alliance, College of Science, Health, Engineering and Education, Murdoch University, Murdoch, WA, Australia; ^4^Department of Botany, College of Life Sciences and Technology, Gansu Agricultural University, Lanzhou, China

**Keywords:** phosphorylation, succinylation, Pi stress, root, corsstalk, barley

## Abstract

Phosphate (Pi) stress is an important environmental factor that limits plant growth and development. Of various posttranslational modifications (PTMs), protein phosphorylation and succinylation are the two most important PTMs that regulate multiple biological processes in response to Pi stress. However, these PTMs have been investigated individually but their interactions with proteins in response to Pi stress remain poorly understood. In this study, to elucidate the underlying mechanisms of protein phosphorylation and succinylation in response to Pi stress, we performed a global analysis of the barley root phosphorylome and succinylome in Pi starvation and recovery stages, respectively. A total of 3,634 and 884 unique phosphorylated and succinylated proteins, respectively, corresponding to 11,538 and 2,840 phospho- and succinyl-sites, were identified; of these, 275 proteins were found to be simultaneously phosphorylated and succinylated. Gene Set Enrichment Analysis was performed with a Kyoto Encyclopedia of Genes and Genomes pathway database revealing pathways that significantly enriched in the phosphorylome and succinylome. Such pathways, were dynamically regulated by Pi starvation and recovery treatments, and could be partitioned into distinct metabolic processes. In particular, phosphorylated proteins related to purine, the mitogen-activated protein kinase (MAPK) signaling pathway, pyrimidine, and ATP-binding cassette (ABC) transporters were upregulated in both Pi deprivation and recovery stages. Succinylated proteins, significantly upregulated by both Pi starvation and recovery, were enriched in nitrogen metabolism and phenylpropanoid biosynthesis. Meanwhile, succinylated proteins that were significantly downregulated by both Pi starvation and recovery were enriched in lysine degradation and tryptophan metabolism. This highlighted the importance of these metabolic pathways in regulating Pi homeostasis. Furthermore, protein–protein interaction network analyses showed that the response of central metabolic pathways to Pi starvation and recovery was significantly modulated by phosphorylation or succinylation, both individually and together. In addition, we discovered relevant proteins involved in MAPK signaling and phenylpropanoid biosynthetic pathways existing in interactions between phosphorylated and succinylated proteins in response to Pi recovery. The current study not only provides a comprehensive analysis of phosphorylated and succinylated proteins in plant responses to Pi starvation and recovery, but also reveals detailed interactions between phosphorylated and succinylated proteins in barley roots.

## Introduction

Proteins play critical roles in essential plant biological processes. Their diversity of functions is regulated by a wide range of posttranslational modifications (PTMs), which are central to the modulation of proteins activity, stability, subcellular localization, and interactions with other functional units (Millar et al., 2019; Willems et al., 2019). In recent decades, based on rapid advances in high-throughput mass spectrometry (MS), more than 461 PTMs have been identified in eukaryotic cells. Thousands of PTM sites can now be comprehensively discovered and quantified in a single proteomics experiment (UniProt Consortium, 2018), including phosphorylation, ubiquitination, sumoylation, glycosylation, acetylation, and succinylation (Willems et al., 2019). Most PTMs of proteins are dynamic, whereby their formation is dependent on the specific targeting of an amino acid residue involving the recognition, addition, or removal of a modification; modular domains are termed a reader, writer, or eraser, respectively (Creixell and Linding, 2012). Furthermore, apart from a single regulatory PTM, multiple PTMs can positively or negatively influence the activities of each other in what is, termed PTM crosstalk (Venne et al., 2014). In plants, such PTMs have been individually investigated in depth among various species; however, studies on PTM crosstalk are only now just emerging.

Phosphorus (P) is an essential mineral macronutrient for plant growth since it is a central component of key molecules such as ATP, nucleic acids, and phospholipids. The roots of plants take up P from soil exclusively in the form of inorganic phosphate (Pi), an ion that is inadequate in sustaining normal plant growth in most agricultural ecosystems due to its low solubility and mobility in soil (Raghothama, 1999; Hinsinger et al., 2011). In fact, the availability of Pi for plants is low, with only about 20% available in applied phosphorus fertilizer. This has aggravated the massive consumption of nonrenewable phosphorus fertilizer resources, causing severe environmental pollution (Pan et al., 2019). To replicate Pi-deficient stress, plants have evolved complex regulatory strategies to improve Pi-acquisition efficiency (i.e., Pi acquisition through the root system), and/or Pi-use efficiency (i.e., Pi remobilization within the plant itself; Vance et al., 2003). In terms of improving Pi-acquisition efficiency, plants modulate their root system architecture by reducing primary roots and increasing lateral root density and root hairs to enlarge the root surface area for Pi uptake in Pi-deficient soils. However, plants also secrete organic acids and enzymes to enhance Pi bioavailability in the rhizosphere soil (Péret et al., 2014).

These well-regulated systems encompass morphological, physiological, biochemical, and molecular adaptations that are controlled by a sophisticated gene regulatory network and are known as the phosphate starvation response (PSR; Chiou and Lin, 2011). In recent years, a growing number of plant studies have revealed that PTM is an important and central regulatory mechanism in the regulation of PSR. Acetylation of histones is essential for the regulation of gene expression for PSRs (Kumar et al., 2021). In Arabidopsis, the histone acetyltransferase, GCN5, positively regulates long non-coding RNA *At4* expression under Pi deficient conditions by modulating its H3K14ac level, resulting in impaired Pi allocation and accumulation in the plant (Wang et al., 2019). Histone deacetylase complex1 (hdc1), involved in the inhibition of primary root growth under Pi deficient conditions, affected the histone H3 acetylation of genes related to the remodeling of root system architecture (Xu et al., 2020). Direct targets of the HDA19 histone deacetylase, complex have not yet been identified. However, several Pi deficiency induced SPX domain containing genes showed decreased expression in HDA19 knock-down *Arabidopsis* lines, including the transcription factors, *SPX3* and *SPX1*. Notably, it is quite possible that HDA19 controls the length of root epidermal cells in response to Pi deficiency by mediating histone PTMs (Chen et al., 2015). Many studies have shown that regulation of ubiquitination is central in the control of PSR in plants, especially in remodeling response of the root system architecture to Pi starvation (Pan et al., 2019). Recently, activation of *Arabidopsis thaliana* plant U box/armadillo repeat-containing E3 ligase9 by receptor kinase2 ubiquitinated the repressor protein of auxin accumulation, and then targeted the autophagy process to improve lateral root development under Pi starvation (Deb et al., 2014). Pi deficiency leads to degradation of the transcription factor, *WRKY6*, mediated by a ubiquitin E3 ligase, PRU1. Ubiquitinated *WRKY6* also significantly accumulated under Pi-deficient conditions, which reduced the expression of *PHO1* to modulate Pi homeostasis (Ye et al., 2018).

Furthermore, the expression of the SPX-domain containing protein, SPX4, is reduced under Pi-starvation conditions. Its degradation is regulated by two RING-finger ubiquitin E3 ligases, SDEL1 and SDEL2. These ligases directly ubiquitinate the K213 and K299 lysine residues in SPX4 to modulate PHR2 activity, thus coordinately regulating Pi signaling and homeostasis in response to Pi stress in rice (Ruan et al., 2019). SUMOylation has received much attention due to it governing Pi homeostatic responses to Pi deficiency. For example, a small ubiquitin-like modifier (SUMO) E3 ligase, SIZ1, which mediates the SUMOylome, plays a pivotal role in the remodeling of root system architecture (Datta et al., 2018; Fang et al., 2021). In addition, results from the global profiling of phosphorylation during Pi starvation in rice roots revealed decreased phosphorylation of the protein kinases, CK2, mitogen-activated protein kinase (MAPK), and calcium-dependent protein kinase (Yang et al., 2019).

Barley (*Hordeum vulgare* L.) is a major cereal crops that is cultivated worldwide. In spite of barley having a strong tolerance to barren soil, its growth and development are seriously affected by Pi deficiency in many areas across the world (Harlan and Zohary, 1966). Resolving barley PTM regulatory mechanisms in response to a Pi deficit will lead to improvements in phosphate acquisition and utilization efficiency in crops. However, compared with other crops, research on PTMs during the phosphate starvation response in barley is still very limited. We have pioneered research on the identification of phosphorylation and lysine succinylation in barley in response to Pi starvation and the recovery process, respectively (Ma et al., 2021; Wang et al., 2021). Although these studies revealed that phosphorylated and succinylated proteins were involved in a wide variety of biological processes, an understanding of PTM-mediated crosstalk between protein phosphorylation and succinylation is still largely unknown in barley.

Distinct from previous reports, in the present study, we describe a comprehensive map of phosphorylation and succinylation dynamics in barley seedling roots in response to Pi-deficient and Pi-replete processes. In this, a Gene Set Enrichment Analysis (GSEA) strategy was used to analyze the features of phosphorylated and succinylated proteins during Pi starvation and recovery stages, with a focus on the cross talk between protein phosphorylation and succinylation, respectively. We successfully identified 275 proteins that were commonly modified by the two PTMs. Both phosphorylated and succinylated proteins showed a distinct difference in metabolic pathways during Pi starvation and the recovery process. Using these resources, we generated a specific metabolic regulatory network for the responses of phosphorylated and succinylated proteins to changing Pi supply. Our findings provide crucial clues for further understanding the cross talk between phosphorylated and succinylated proteins in response to Pi starvation in plants.

## Materials and methods

### Plant materials and treatments

**Figure 1:**
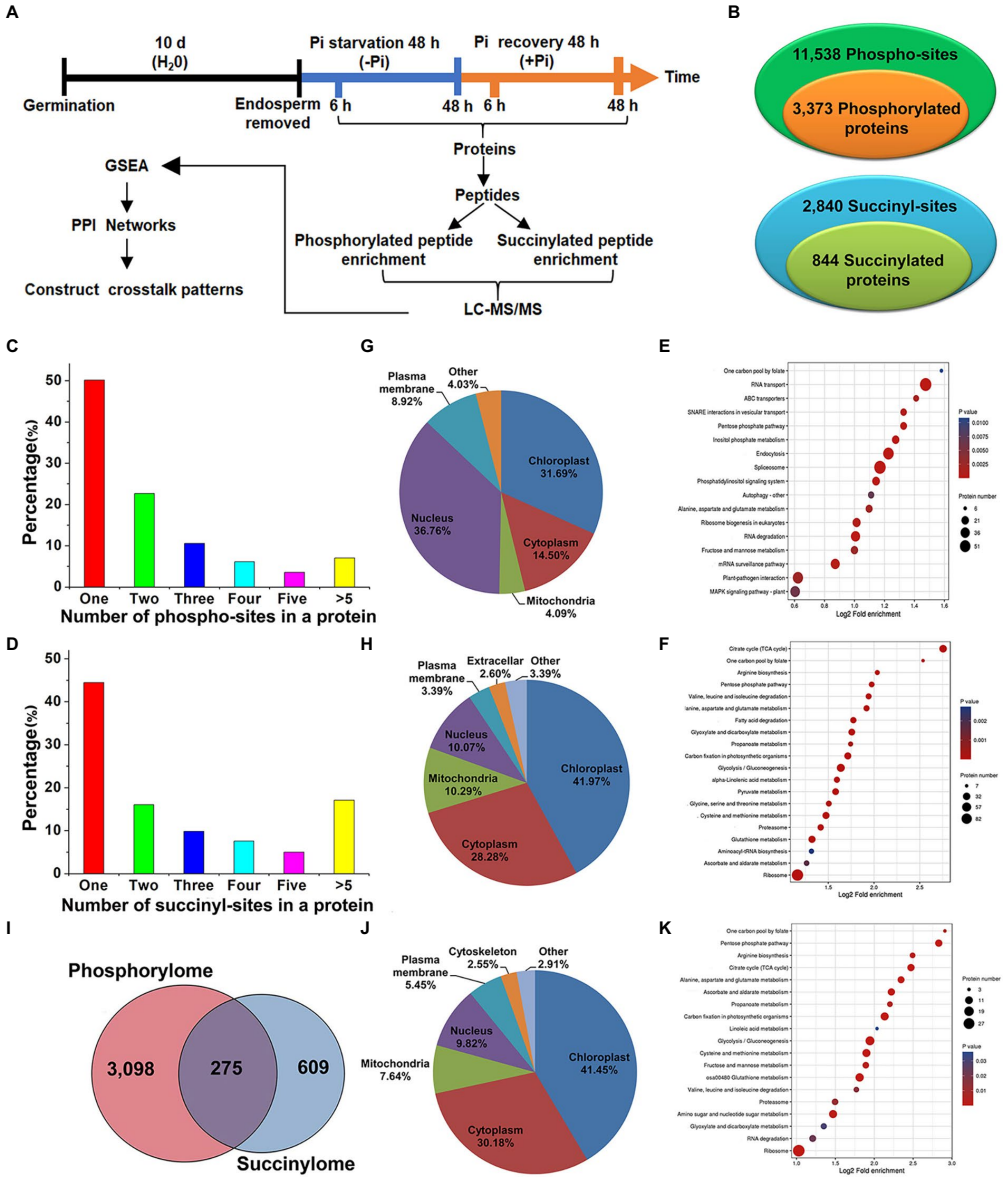
Experimental workflow and profiling phosphoproteome and succinylome responses of barley roots to Pi starvation and recovery. The workflow of the integrated analysis of phosphoproteome and succinylome data **(A)**. Detection of phosphorylated and succinylated proteins **(B)**. The number of phospho- and succinyl-sites within phosphorylated **(C)** and succinylated **(D)** proteins, respectively. Subcellular localization and KEGG pathway enrichment analysis of phosphorylated (**G,E**) and succinylated proteins (**H,F**), respectively. Overlap of phosphorylated and succinylated proteins (**I**). Subcellular localization and KEGG pathway enrichment analysis of both phosphorylated and succinylated proteins (**J,K**), respectively. GSEA, Gene Set Enrichment Analysis; KEGG, Kyoto Encyclopedia of Genes and Genomes; LC–MS/MS, liquid chromatography with tandem mass spectrometry; Pi, intracellular phosphate; PPI, protein–protein interaction.

The design of this study references the transcriptome analysis of a rice root response to Pi stress starvation and recovery by Secco et al. (2013) and is shown in Figure 1A. In our previous study, a low-Pi tolerant barley (*Hordeum vulgare* L.) genotype GN121 was identified. The roots of this genotype differ in how their architecture changes to respond to Pi starvation compared with low-Pi sensitive GN42 (Ren et al., 2016; Wang et al., 2021). GN121 was used in this present work. Seed germination, growth conditions, and Pi-stress treatment of GN121 plants were as previously described (Ren et al., 2018). Briefly, GN121 seeds without residual endosperm uniformly germinated for 10 days then transferred to modified Hoagland hydroponic nutrient solution with 0.39 mM KH_2_PO_4_ (high Pi, +Pi) or 0.039 mM KH_2_PO_4_ (low Pi, –Pi) as the only Pi source. For Pi starvation and re-supply, plants were grown under –Pi for 48 h (Pi-starvation process) and then resupplied with +Pi for 48 h (Pi-recovery process). Roots were harvested under Pi-starvation and Pi-recovery processes after 6 h and 48 h for three biological replicates, respectively. Six roots were randomly collected for each biological replicate. The root samples were frozen in liquid nitrogen and stored at −80°C for protein extraction.

### Proteomics analysis

Further details on protein extraction, digestion, phosphopeptide and succinyl-peptide enrichment, liquid chromatography with tandem mass spectrometry (LC–MS/MS) analysis, and database searching are outlined in the our previously articles (Ma et al., 2021; Wang et al., 2021). In summary, for phosphopeptide enrichment, tryptic peptides were dissolved in a 6% (v/v) TFA/50% ACN (v/v) buffer and the supernatant was incubated with an immobilized metal affinity column that bound phosphopeptides. To enrich for succinyl-peptides, tryptic peptides were dissolved in immunoprecipitation buffer and the supernatant was incubated with pre-washed antibody beads (PTM402; PTM Biolabs, Hangzhou, China) that bound succinyl-peptides. Dissolved samples were subsequently injected into an EASY-nLC 1,000 ultraperformance liquid chromatography system (Thermo Fisher Scientific, Waltham, MA, United States) and loaded onto an in-house reversed-phase analytical column (15-cm length, 75 μm i.d.). The electrospray voltage applied was 1.6 kV. Precursors and fragments were detected in a TOF detector, with a m/z scan range from 100 to 1700. Parallel accumulation serial fragmentation (PASEF) mode was used for the primary MS acquisition, and 10 times PASEF-MS/MS scans were acquired 1 cycle with the charge states in the range of 0–5. The dynamic exclusion time was set to 30 s. The resulting MS/MS data were processed using a MaxQuant search engine (v.1.6.6.0). Finally, tandem mass spectra were searched against a *Hordeum vulgare* L. protein database^1^ (39,743 protein entries). False discovery rate thresholds for protein, peptide, and modification sites were adjusted to <1% and the minimum score for modified peptides was set to >40. Label-free, intensity-based, absolute quantification values in MaxQuant were used to quantify phosphorylated and succinylated protein abundance (Cox et al., 2014). The mass spectrometry profiles of proteome, phosphoproteome, and succinyl-proteome data are available *via* ProteomeXchange with the identifiers PXD022052, PXD022077 and PXD022053, respectively.

### Bioinformatics analysis

The overall aims of this study were to compare different quantified phospho- and succinyl-proteins, and, further, to reveal cross talk patterns between such quantified proteins. First, phospho- and succinyl-proteins were assigned to functional categories using a Kyoto Encyclopedia of Genes and Genomes (KEGG) pathway database. Then, a threshold-free method, Gene Set Enrichment Analysis (GSEA) was used to assess metabolic pathways that were significantly and differentially expressed during Pi-starvation and Pi-recovery processes for individual quantified phospho- and succinyl-proteins, as well as both quantified phospho- and succinyl-proteins, using GSEA software, respectively (Subramanian et al., 2005). Five or more genes were allowed in each set, and the ranked genes were used as inputs to GSEAPreRanked with default options except that gene set permutations were performed 1,000 times. Metabolic pathways with *p*-values less than 0.05 for the permutation test were defined as significant differentially expressed metabolic pathways. The subcellular localization data of proteins was obtained from a Eukaryotes database of Wolfpsort^2^ based on annotations (Horton et al., 2007). For the identification of similarly regulated phosphoryl- and succinyl-proteins, expression profile clustering at four different time points was performed using a Mfuzz package (v2.32.0) for R programming language (Kumar and Futschik, 2007). Briefly, the input table of mean normalized protein intensity values was organized into columns with separate rows for each protein. We used a fuzzy c-means (FCM) clustering algorithm; FCM assigns a membership value to each profile in the range [0,1] for each of the c clusters. The final clustering was done with the parameters *c* = 6 and *m* = 1.5. Finally, protein–protein interaction (PPI) networks for identified proteins showing phosphorylation, succinylation and both phosphorylation and succinylation, were obtained from STRING software (v.11.0) and visualized by Cytoscape (3.7.2) software by applying a confidence score of 0.4 (Shannon et al., 2003).

### Coimmunoprecipitation and MS/MS analysis

We performed a coimmunoprecipitation (co-IP) assay and MS/MS analysis to further validate protein phosphorylation and succinylation results. Coimmunoprecipitation experiments were performed according to the manufacturer’s protocol using phospho-serine (Cat. #3192) and succinylated lysine (Cat. #3089) polyclonal antibodies from Dia-an Biotechnology Incorporation (Wuhan, China). Briefly, roots of GN121 in Pi starvation for 6 h and 48 h, and in Pi recovery for 6 h and 48 h, as described above, were frozen in liquid nitrogen and ground to a fine powder. The powder was lysed for 30 min in ice-cold western blot/immunoprecipitation buffer (Beyotime, Shanghai, China) containing protease and phosphatase inhibitors. Samples were then centrifuged for 10 min at 15,000 × *g* at 4°C, and supernatants transferred to new tubes. Each supernatant was then precleared by incubation with 10 μl of phospho-serine or succinylated lysine polyclonal antibody and 50 μl of protein A/G magnetic beads for 1 h at room temperature, followed by centrifugation and the supernatant discarded. The protein A/G magnetic beads were washed three times with phosphate buffered saline (PBS), and cell lysates (200 μl) were incubated with the corresponding antibody at 4°C overnight. Finally, each pellet was resuspended in 50 μl PBS buffer after washing with PBS three times, and a 20-μl sample was used and subsequently analyzed by western blotting. Western blotting experiments were based on previously described methods by Zeng et al. (2021). The primary antibodies used in the western blot were phospho-serine polyclonal antibody (1,500 in TBST with 5% nonfat milk), and succinylated lysine polyclonal antibody (1,300 in TBST with 5% nonfat milk) incubated overnight at 4°C. A goat anti-rabbit antibody with conjugated horse radish peroxidase was used as a secondary antibody at a 1:5000 dilution in TBST with 3% nonfat milk. The immunoprecipitated proteins eluted from supernatants were used for identification by liquid chromatography (LC)–MS/MS as described above.

## Results

### Global characterization of protein phosphorylation and succinylation

To compile a comprehensive protein phosphorylation and succinylation profile of barley roots in response to Pi starvation and recovery, we performed a time-course experiment involving Pi deprivation and resupply of Pi-starved plants for up to 48 h. In total, four time points, Pi deprivation at 6 h and 48 h, and Pi resupply at 6 h and 48 h, were selected to assess protein phosphorylation and succinylation changes in roots (Figure 1A). Our sequential affinity enrichment workflow identified 3,373 and 884 unique phosphorylated and succinylated proteins corresponding to 11,538 and 2,840 phospho- and succinyl-sites, respectively (Figure 1B; Supplementary Table S1). The 3,373 phosphoryl-proteins and 884 succinyl-proteins that were identified accounted for 53.97 and 13.12%, respectively, of the total number of identified barley root proteins.

We next evaluated the distribution of phosphorylation and succinylation sites identified on proteins by counting the number of modification sites. Of the 3,634 detected phosphorylated proteins, those proteins with one, two, three, four, five or more phosphorylation sites comprised 50.13, 22.65, 10.55, 6.11, 3.56, and 7.00% of modified protein sites, respectively (Figure 1C). A pSer modification comprised 73.29%, pThr 24.46%, and pTyr is 2.25% of modified protein sites, respectively (Supplementary Table S1). Of the 884 succinylation proteins detected, about 44.46% contained a single lysine succinylation site. Proteins with two, three, four, five or more succinylated sites comprised 16.06, 9.84, 7.58, 4.98, and 17.08% of modified protein sites, respectively (Figure 1D). Pathway enrichment analysis revealed that proteins modified by phosphorylation and succinylation were involved in distinct metabolic processes although several processes existed in which both PTMs were over-represented. In particular, we found protein phosphorylation predominantly on proteins related to RNA transport, the spliceosome, endocytosis, RNA degradation, and plant pathogen interactions (Figure 1E; Supplementary Table S2), while succinylation occurred on proteins involved in the ribosome, tricarboxylic acid (TCA) cycle, and glycolysis/gluconeogenesis (Figure 1F; Supplementary Table S3). Subcellular location profiles showed that approximately 68.5% of all phosphorylated proteins were located in the nucleus and chloroplasts (Figure 1G). This indicates that the roots may regulate intra-nuclear processes and chloroplast protein functions through the phosphorylation of relevant proteins. Similarly, about 70.25% of all succinylated proteins were located in the cytoplasm and chloroplasts (Figure 1H), which suggests that protein succinylation has a critical role in regulating extensive cytosolic processes. To obtain more detailed information on co-occurring proteins modified by both phosphorylation and succinylation, we compared all identified phosphorylation and succinylation events occurring on proteins. We showed that only 275 proteins were both phosphorylated and succinylated (Figure 1I). Approximately 72.0% of these proteins were located in the cytoplasm and chloroplasts (Figure 1J). Functions related to the ribosome, glycolysis/gluconeogenesis, and glutathione metabolism, especially in ribosome processes, were significantly enriched (Figure 1K; Supplementary Table S4). Taken together, these analyses suggest that protein phosphorylation and succinylation were frequently occurring PTMs that might have essential regulatory roles in the response of barley roots’ to Pi stress.

### Profiling protein phosphorylation and succinylation involved in responses to Pi stress

**Figure 2:**
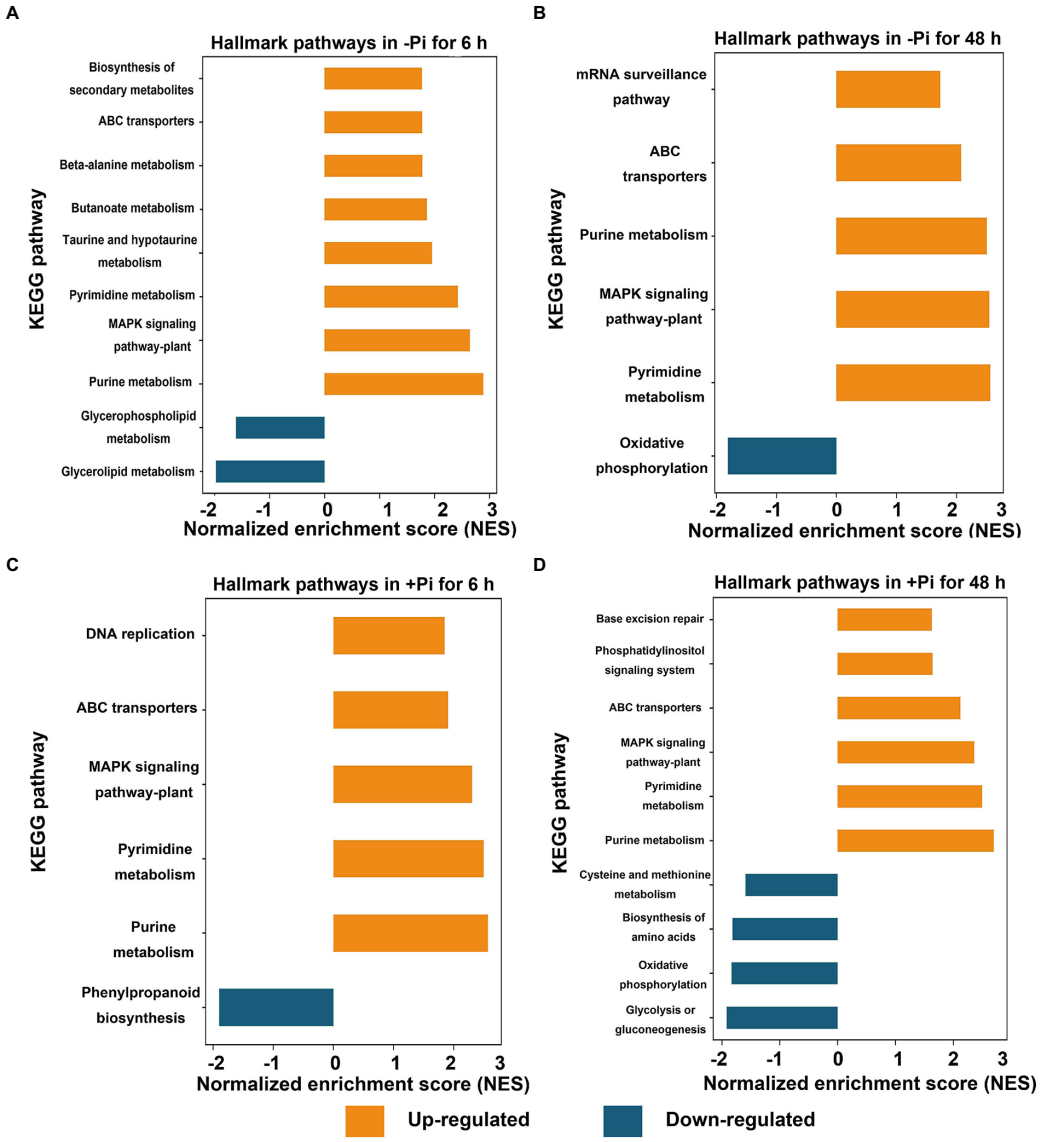
GSEA of significantly up (FDR < 0.1, NES > 1.5; orange) or down (FDR < 0.1, NES < –1.5; blue) regulated metabolic pathways in response to Pi starvation (–Pi) for 6 h **(A)** and 48 h **(B)** and Pi recovery (+Pi) for 6 h **(C)** and 48 h **(D)** of phosphorylated proteins, respectively. Gene sets with an FDR < 0.01 are displayed using bar plots. FDR, false discovery rate; GSEA, Gene Set Enrichment Analysis; NES, normalized enrichment score; Pi, intracellular phosphate.

**Figure 3:**
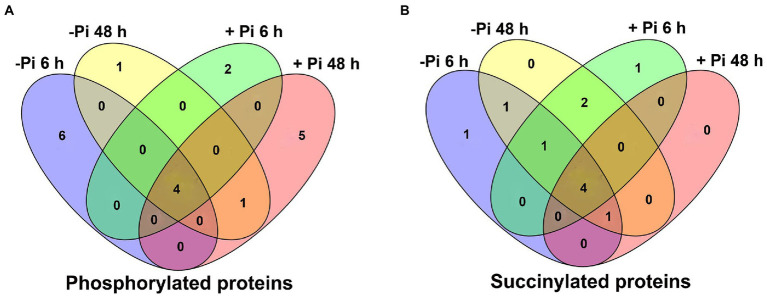
Venn diagram representing the overlap of differentially expressed metabolic pathways in Pi starvation (–Pi) for 6 h and 48 h, and Pi recovery (+Pi) for 6 h and 48 h with regard to phosphorylated **(A)** and succinylated **(B)** proteins, respectively. Pi, intracellular phosphate.

To identify differentially expressed metabolic pathways in phosphoryl- and succinyl-proteomes in response to Pi starvation, we employed GSEA to determine the significance of a change in protein expression during Pi deficit and recovery at different time points relative to the control sample. The GSEA of KEGG pathway analysis revealed that both Pi deficit and recovery were enriched in phosphoryl- and succinyl-proteomes in response to Pi stress and could be partitioned into distinct metabolic processes. However, two processes (MAPK signaling, and phenylpropanoid biosynthetic pathways) existed in which both PTMs were over-represented (Figures 2, 3; Supplementary Tables S5–S12). With regard to phosphorylome data, after 6 h of Pi deprivation, proteins related to purine metabolism, the MAPK signaling pathway, pyrimidine metabolism, and another five pathways were significantly upregulated, while proteins belonging to glycerolipid and glycerophospholipid metabolism were significantly downregulated (Figure 2A; Supplementary Table S5). Proteins involved in pyrimidine metabolism, the MAPK signaling pathway, purine metabolism, ABC transporters, and the mRNA surveillance pathway were upregulated, and those involved in oxidative phosphorylation were downregulated after 48 h of Pi deprivation (Figure 2B; Supplementary Table S6). Similarly, during Pi resupply for 6 h, GSEA of KEGG pathway enrichment revealed that proteins that were involved in DNA replication, ABC transporters, the MAPK signaling pathway, and pyrimidine, and purine metabolism were significantly upregulated, while phenylpropanoid biosynthesis was significantly downregulated (Figure 2C; Supplementary Table S7). Proteins belonging to purine and pyrimidine pathway, the MAPK signaling pathway, and ABC transporters were significantly upregulated, and glycolysis of gluconeogenesis, oxidative phosphorylation, and biosynthesis of amino acids were significantly downregulated under Pi resupply for 48 h, respectively (Figure 2D; Supplementary Table S8). In particular, we observed that of these enrichment pathways, only proteins related to purine and pyrimidine metabolism, the MAPK signaling pathway, and ABC transporters were upregulated in either Pi deprivation and/or recovery stages, respectively (Figure 3A).

**Figure 4:**
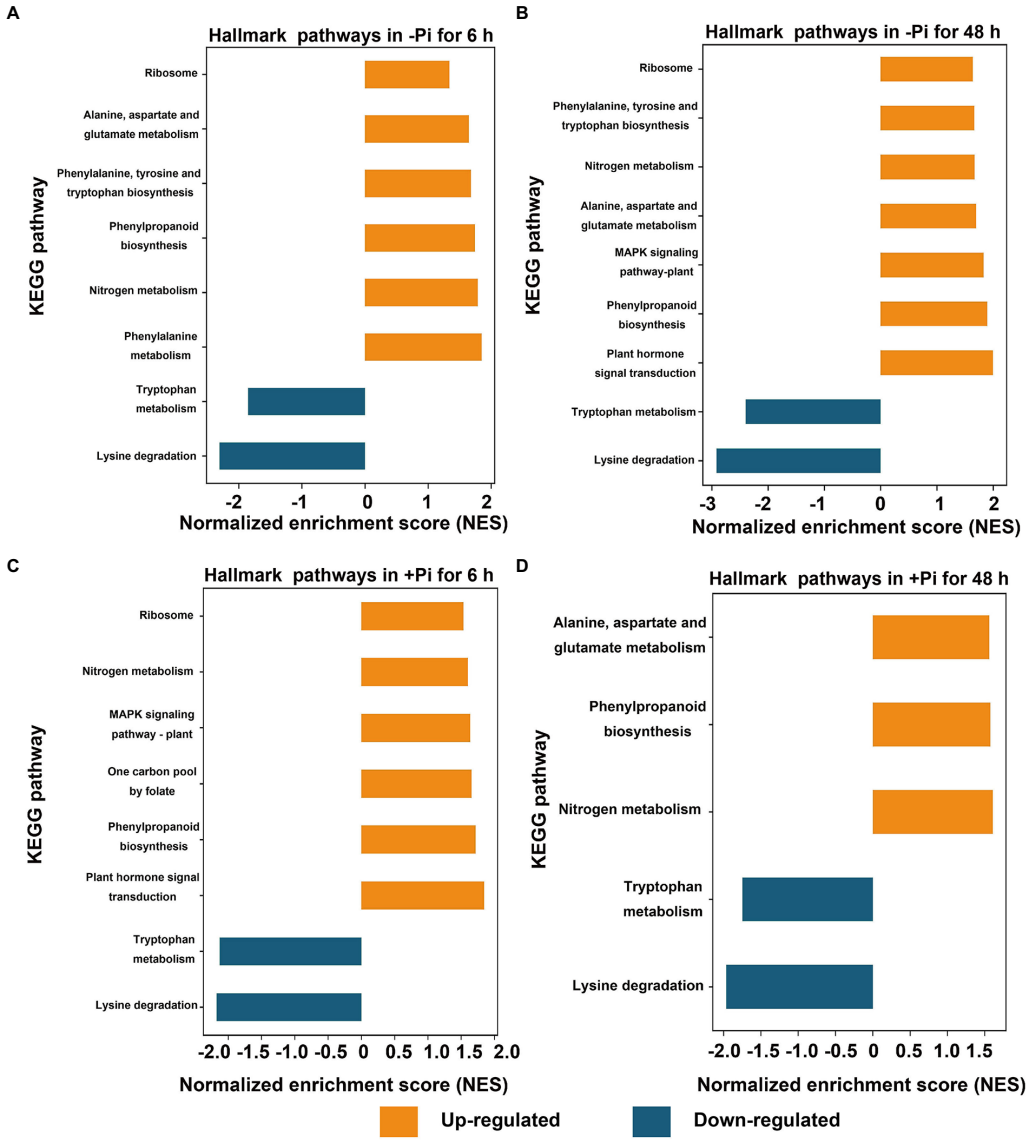
GSEA of significantly up- (FDR < 0.1, NES > 1.5; orange) or down- (FDR < 0.1, NES < –1.5; blue) regulated metabolic pathways in response to Pi starvation (–Pi) for 6  h **(A)** and 48 h **(B)**, and Pi recovery (+Pi) for 6 h **(C)** and 48 h **(D)** with regard to succinylated proteins, respectively. Gene sets with an FDR < 0.01 are displayed using bar plots. FDR, false discovery rate; GSEA, Gene Set Enrichment Analysis; NES, normalized enrichment score; Pi, intracellular phosphate.

Furthermore, for succinylome data, in a GSEA analysis of Pi deficiency for 48 h, we found that proteins involved in phenylalanine, nitrogen metabolism, phenylpropaneoide biosynthesis, phenylalanine, tyrosine and tryptophan biosynthesis, alanine, aspartate and glutamate metabolism, and the ribosome metabolic pathway were significantly upregulated, and lysine degradation and tryptophan metabolism were downregulated during Pi starvation, respectively (Figure 4A; Supplementary Table S9). In Pi deficiency for 48 h, proteins related to plant hormone signal transduction, phenylpropanoid biosynthesis, the MAPK signaling pathway, alanine, aspartate and glutamate metabolism, nitrogen metabolism, phenylalanine, tyrosine and tryptophan biosynthesis, and ribosomes were significantly upregulated, while those associated with lysine degradation and tryptophan metabolism were downregulated (Figure 4B; Supplementary Table S10). Under Pi recovery for 6 h, proteins belonging to plant hormone signal transduction, phenylpropanoid biosynthesis, one carbon pool by folate pathway, the MAPK signaling pathway, nitrogen metabolism, and ribosomes were significantly upregulated, while proteins related to lysine degradation and tryptophan metabolism were significantly downregulated, respectively (Figure 4C; Supplementary Table S11). After 48 h of Pi recovery, proteins in nitrogen metabolism, phenylpropanoid biosynthesis, and alanine, aspartate and glutamate metabolism were upregulated, while those related to lysine degradation and tryptophan metabolism were downregulated significantly (Figure 4D; Supplementary Table S12). Intriguingly, proteins significantly downregulated by both Pi starvation and/or recovery were enriched in lysine degradation and tryptophan metabolism, with both belonging to amino acid metabolism. Meanwhile, proteins that were significantly upregulated by both Pi starvation and/or recovery were enriched for nitrogen metabolism and phenylpropanoid biosynthesis (Figures 3B, 4).

### Expression pattern of phosphoryl- and succinyl-proteins in response to Pi stress

**Figure 5:**
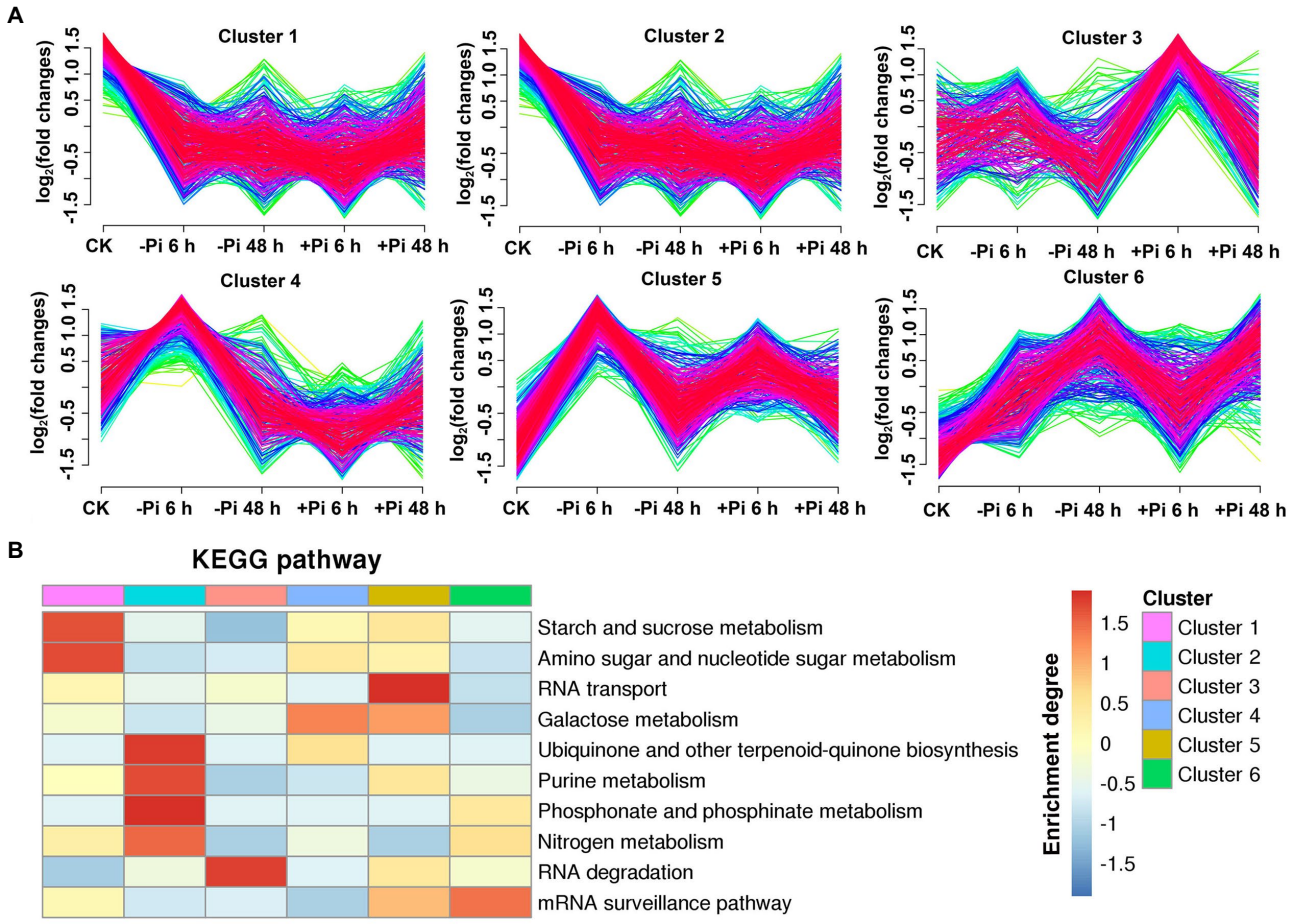
Clustering analysis of phosphorylated proteins based on their expression pattern in response to Pi starvation and recovery using a Mfuzz package **(A)**; and KEGG pathways that are overrepresented in each cluster based on the relative phosphorylation intensity relative to control **(B)**. –Pi, Pi starvation; +Pi, Pi recovery. The colored scale bar shows the enrichment degree of pathways in order perform z-score processing on-log (Fisher’s exact test *p* value). KEGG, Kyoto Encyclopedia of Genes and Genomes; Pi, intracellular phosphate.

**Figure 6:**
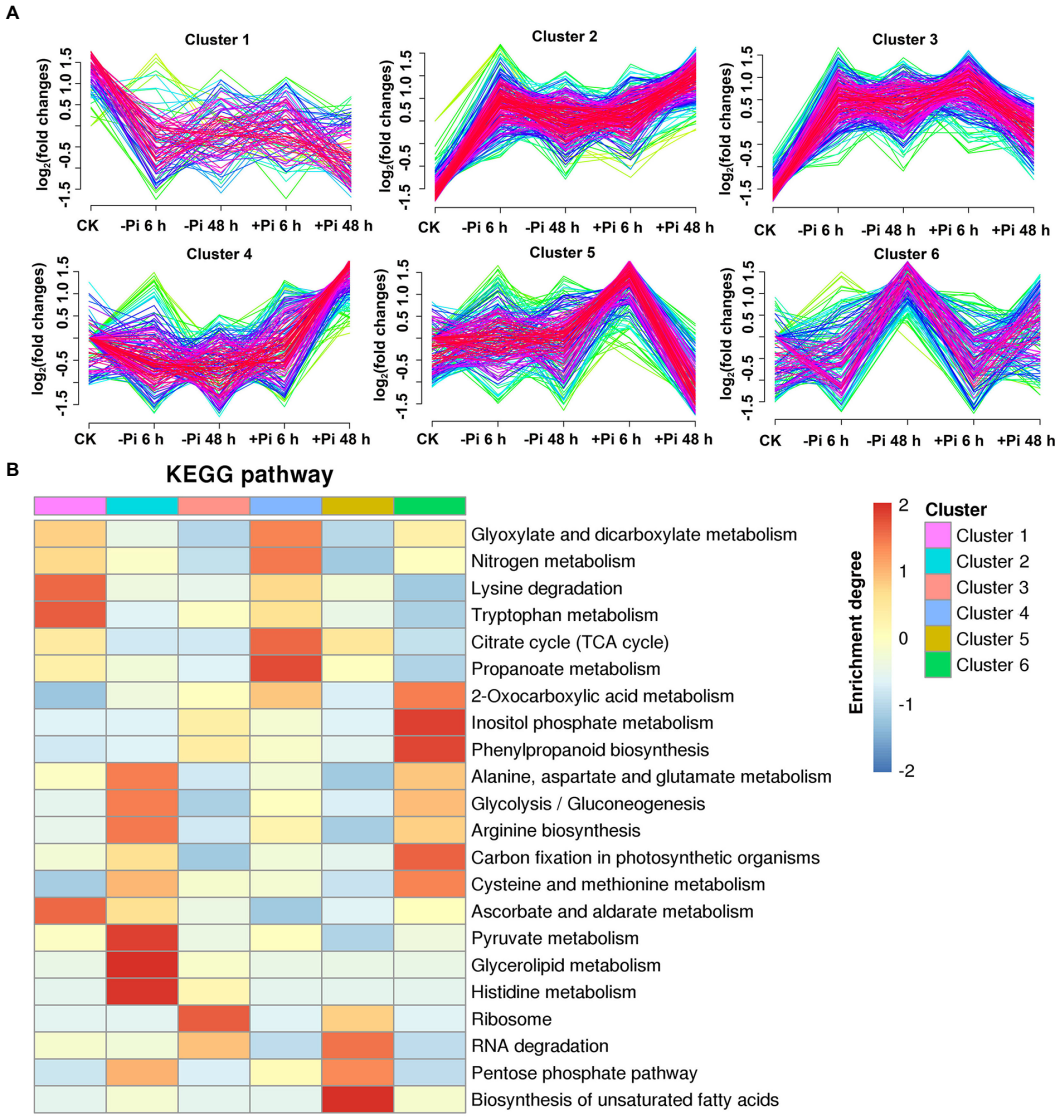
Clustering analysis of succinylated proteins based on their expression pattern in response to Pi recovery using a Mfuzz package **(A)** and KEGG pathways that are overrepresented in each cluster based on the succinylation intensity relative to the control **(B)**. –Pi, Pi starvation; +Pi, Pi recovery. The colored scale bar shows the enrichment degree of pathways to perform *z*-score processing on-log (Fisher’s exact test *p* value). KEGG, Kyoto Encyclopedia of Genes and Genomes; Pi, intracellular phosphate.

To explore the characteristics of phosphoryl- and succinyl-proteins in response to the processes of Pi starvation and then Pi resupply, we classified mainly phosphoryl- and succinyl-proteins into six groups according to their expression patterns in response to Pi treatments (Figures 5A, 6A). From the perspective of protein expression patterns, phosphoryl- and succinyl-proteins showed different response patterns to Pi starvation and recovery. At the same time, it was difficult to determine the protein sets that specifically responded to Pi starvation and recovery. This may have been due to short-term Pi starvation and recovery stages that were insufficient to induce specific Pi stress perception and signal transduction by phosphoryl- and succinyl-proteins. For phosphoryl-proteins, cluster 2 and 4 proteins showed a response to Pi starvation but were not responsive to Pi recovery; these proteins were enriched in starch and sucrose metabolism, urine metabolism, and nitrogen metabolism (Figure 5B; Supplementary Table S13). Cluster 1, 3, 5, and 6 proteins responded to both Pi starvation and resupply; these proteins were mainly enriched in RNA transport, and RNA degradation, among other pathways. This was especially so for proteins belonging to cluster 6 that were continuously upregulated in Pi starvation, and that were enriched in the mRNA surveillance pathway (Figure 5B; Supplementary Table S13).

For succinyl-proteins, cluster 2 proteins responded to Pi starvation and persisted in their response during Pi resupply; proteins belonging to this class were enriched in the TCA cycle, glycolysis/gluconeogenesis, and pyruvate metabolism, among other pathways (Figure 6B; Supplementary Table S14). Cluster 4 and 5 proteins showed a response to Pi recovery but were not responsive to Pi starvation, including those in the response to glycolysis/gluconeogenesis, glyoxylate and dicarboxylate metabolism, TCA cycle, and ribosome pathway. Cluster 1, 3, and 6 proteins responded to both Pi starvation and recovery; such proteins were mainly enriched in the TCA cycle, glyoxylate and dicarboxylate metabolism, ribosome, glycolysis/gluconeogenesis, carbon fixation in photosynthetic organisms, and cysteine and methionine metabolism, among other pathways (Figure 6B; Supplementary Table S14).

### Crosstalk of phosphorylome and succinylome in response to Pi stress

**Figure 7:**
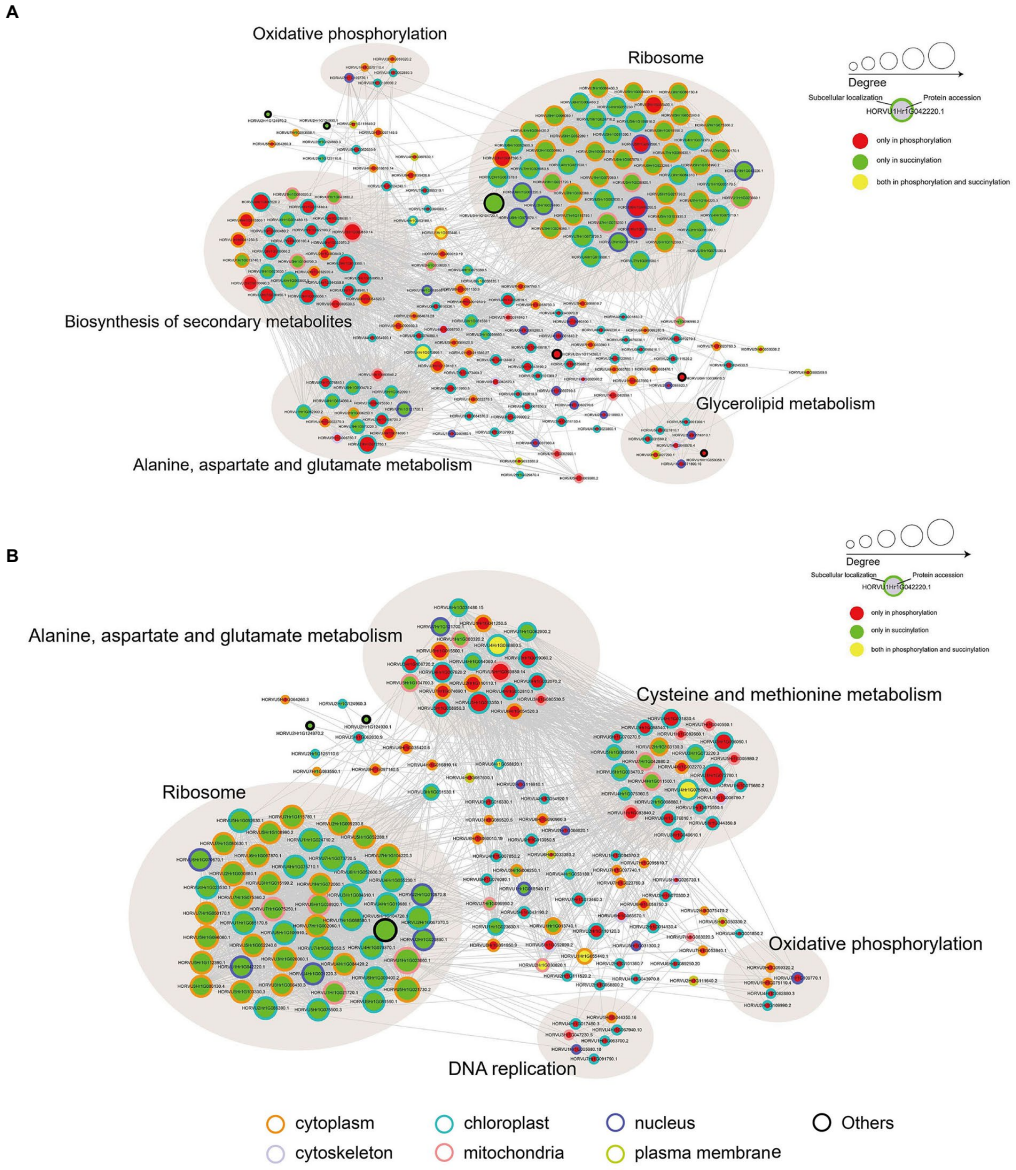
Protein–protein interaction (PPI) network analysis of significantly phosphorylated or succinylated, and both phosphorylated and succinylated proteins in response to Pi starvation **(A)** and recovery **(B)**, respectively. Phosphorylated and succinylated proteins belonging to the significantly enriched KEGG pathway in Pi starvation and Pi recovery stages were used for PPI analysis, respectively. PPI network were obtained from STRING software (v.11.0) and visualized by Cytoscape (3.7.2) after applying a confidence score of 0.4. Light brown circles depict clusters of proteins involved in specific metabolic pathways. The circle size represents the number of interaction nodes; the greater the number of interaction nodes, the larger the circle. Node outlines indicate the predicted subcellular localization of proteins. Cytoplasm (orange), cytoskeleton (lavender), mitochondria (pink), nucleus (purple), plasma membrane (green), chloroplast (blue), and others (black). Further details are in Supplementary Tables S16, S17. KEGG, Kyoto Encyclopedia of Genes and Genomes; Pi, intracellular phosphate; PPI, protein–protein interaction.

To investigate how plant metabolism maybe regulated by phosphorylation and succinylation, we also compared all protein enrichment pathways found to be modified by these PTMs in Pi starvation and recovery processes. Surprisingly, while 275 proteins were shown to undergo both phosphorylation and succinylation events under Pi stress, only two pathways, MAPK signaling and phenylpropanoid biosynthesis pathway, were enriched at the same stress time point of 6 h under Pi resupply (Figures 2, 4). More specifically, for the MAPK signaling pathway, a total of six and two proteins modified by phosphorylation and succinylation, respectively, were identified as core enrichment proteins. Of these, only one protein, HORVU1Hr1G055440.1 (a nucleoside diphosphate kinase family protein) was modified by both phosphorylation and succinylation (Supplementary Table S15). For the phenylpropanoid biosynthesis pathway, nine and four proteins were identified as core enrichment proteins with modified phosphorylation and succinylation, respectively; none of these were modified by both phosphorylation and succinylation simultaneously (Supplementary Table S15). Thus, this finding indicates that the co-occurrence of phosphorylation and succinylation happens not only on different proteins involved in the same pathway, but also on the same proteins at different sites. Finally, to further analyze the crosstalk between both phosphoryl- and succinyl-proteins in Pi starvation and recovery, we constructed PPI networks of both such proteins under Pi starvation and recovery stages, respectively, using Cytoscape software. From these data sets, we found that this crosstalk between phosphoryl- and succinyl-proteins in response to Pi starvation and recovery were highly dynamic and involved in special metabolic processes. During Pi starvation, no metabolic pathways containing both phosphoryl- and succinyl-proteins were enriched. However, Pi resupply resulted in the enrichment of both phosphoryl- and succinyl-proteins associated with amino acid metabolism, such as alanine, aspartate and glutamate metabolism, and cysteine and methionine metabolism (Figure 7; Supplementary Tables S16, S17).

**Figure 8:**
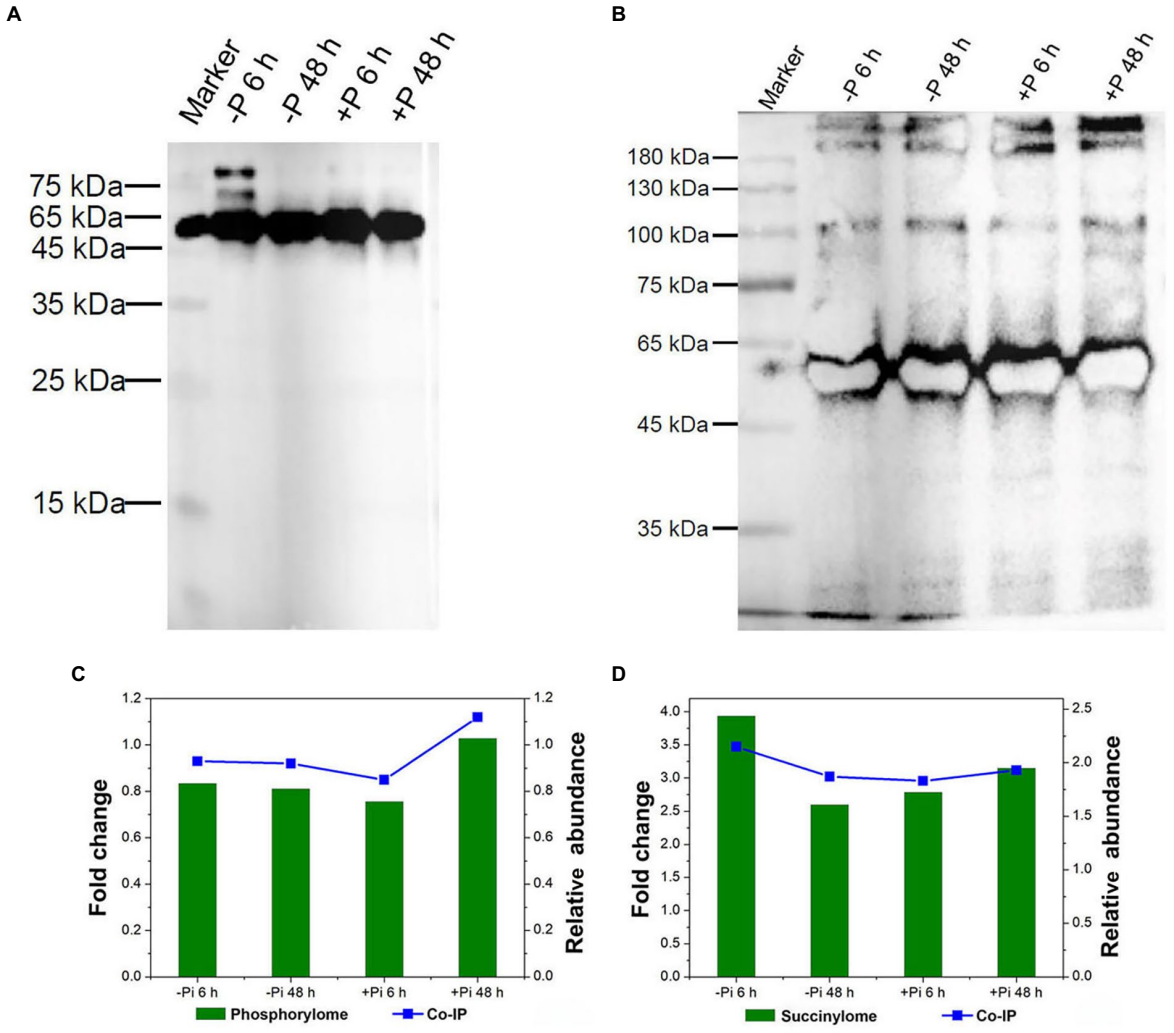
Validation of serine phosphorylation and lysine succinylation proteins in Pi starvation (–Pi) for 6 h and 48 h, and Pi recovery (+Pi) for 6 h and 48 h. Serine phosphorylation **(A)** and lysine succinylation **(B)** proteins were enriched by co-IP with phospho-serine and succinylated lysine polyclonal antibodies, respectively, followed by western blotting. Comparison of the phosphorylation **(C)** and succinylation **(D)** level of HORVU1Hr1G055440.1 in proteomic data (left Y-axis) and co-IP experiments (right Y-axis). Co-IP, co-immunoprecipitation; Pi, intracellular phosphate.

To validate their serine phosphorylated and lysine succinylated status, target proteins were enriched using phospho-serine and succinylated lysine polyclonal primary antibodies and visualized *via* western blotting (Figure 8). Co-immunoprecipitation followed by LC–MS/MS analysis were used to identify serine phosphorylated and lysine succinylated proteins. Finally, a large number of serine phosphorylation and lysine succinylation events were successfully detected in target proteins within the immunoprecipitated samples, respectively (Figures 8A,B). In particular, HORVU1Hr1G055440.1, modified by both phosphorylation and succinylation according to proteomics analysis, exhibited co-modification with phosphorylation and succinylation according to LC–MS/MS analysis. The changes in phosphorylation and succinylation levels were consistent with phosphorylated and lysine succinylated proteomic data (Figures 8C,D), meaning our proteomics analysis of phosphorylation and succinylation results were reliable.

## Discussion

Our current understanding of Pi starvation and recovery in plants is largely derived from gene expression, transcriptome, and proteome studies. These have revealed a large number of potential key regulators of Pi homeostasis in plants, especially in *Arabidopsis thaliana* (Thibaud et al., 2010; Woo et al., 2012) and rice (Secco et al., 2013; Gho et al., 2018). Protein phosphorylation and succinylation are the two most important PTMs for regulating multiple biological processes in plants (Nakagami et al., 2010; Xia et al., 2022). The recent developments of high-resolution mass spectrometry, antibody based affinity enrichment proteomic technology, and powerful bioinformatic tools have substantially contributed to the global analysis of protein phosphorylation and succinylation in barley. In our study in the last year, a large number of phosphoryl- and succinyl-proteins with diverse biological functions were identified to be responsive to Pi stress and recovery in barley roots (Ma et al., 2021; Wang et al., 2021). Although these studies contributed to our understanding of the mechanisms involved in responses to Pi starvation and recovery, they were limited to phosphoryl- and succinyl-proteins that were characterized separately, thus failing to capture any global crosstalk between phosphorylation and succinylation in the regulation of Pi homeostasis. In addition, such studies on phosphoryl- and succinyl-protein pathway enrichment were analyzed by a ranked differential protein list filtered by a particular threshold with a *p* value < 0.05 and fold-change > 1.5 (Ma et al., 2021; Wang et al., 2021), which is more dependent on differential proteins and has a certain subjectivity (Reimand et al., 2019). Here, in order to overcome the above defects, we used a threshold-free, GSEA approach to explore the underlying mechanisms by which protein phosphorylation and succinylation were involved in responses to Pi starvation and recovery in barley roots. These results provide more comprehensive insights into phosphorylation and succinylation responses to Pi stress.

Phosphorylation plays a prominent role in regulating cellular signaling, whereas succinylation is the primary mechanism for coordinating metabolism and cellular signaling (Swaney et al., 2013; Wu et al., 2019). In this study, a total of 3,373 and 884 unique phosphorylated and succinylated proteins, corresponding to 11,538 and 2,840 phospho- and succinyl-sites, were identified by a thorough investigation conducted on the response of barley roots Pi starvation and recovery, respectively (Figure 1B). We compared the KEGG enrichment of phosphorylated and succinylated proteins. Phosphorylated proteins were mainly localized in the nucleus and chloroplasts. An analysis of KEGG enrichment showed that these phosphorylated proteins were involved in processes such as RNA transport, spliceosome, endocytosis, RNA degradation, and plant pathogen interactions. In contrast, succinylated proteins were principally localized in the cytoplasm and chloroplasts, and enriched pathways were mainly involved with ribosomes, the TCA cycle, and glycolysis/gluconeogenesis (Figure 1). Furthermore, relatively minor overlapping was observed between our phosphorylated and succinylated proteins (Figure 1I). Similar results were obtained for the succinyl and acetyl proteomes of rice leaves (Zhou et al., 2018), and the protein phosphorylation and acetylation of proteins in Arabidopsis organs and seedlings (Uhrig et al., 2019). Such proteins, modified by both phosphorylation and succinylation, were mainly enriched in the cytoplasm and chloroplasts, and predominated in the ribosome pathway (Figure 1J). This suggests that proteins found co-occurring with phosphorylation and succinylation are more likely to be functionally important in protein synthesis in response to Pi stress.

To determine how differences in phosphorylation and succinylation of proteins in roots were represented in response to Pi starvation and recovery, we employed GSEA to identify differentially expressed metabolic pathways. Gene Set Enrichment Analysis revealed the presence of 10, 6, 6, and 10 metabolic pathways differentially expressed under Pi starvation for 6 h and 48 h, and under Pi recovery for 6 h, and 48 h, respectively (Figure 2). Of these pathways, purine, the MAPK signaling pathway, pyrimidine, and ABC transporters were upregulated by enrichment at all time points (Figure 2). It was found that purine metabolism played an important role in the acclimatization of *Arabidopsis* to drought (Watanabe et al., 2010; Itam et al., 2020), and pyrimidine metabolites showed an increasing trend under drought-stress conditions in bread wheat (Itam et al., 2020). The MAPK signaling pathway is a part of the complex signaling network for numerous environmental factors as well as plant growth and development. It is usually activated in response to various abiotic stresses, including nutrient status (Danquah et al., 2014; Kumar et al., 2020). ATP-binding cassette transporters participate in diverse biological processes to copy biotic and abiotic stresses. In Arabidopsis, *ALS3* and its interacting protein, *AtSTAR1*, form an ABC transporter complex, which involves the Pi deficiency induced remodeling of RSA by modulation of Fe homeostasis in roots (Dong et al., 2017). Similarly, eight, nine, eight and five differentially expressed metabolic pathways in succinylation proteins were enriched under Pi starvation for 6 h and 48 h, and Pi recovery for 6 h and 48 h, respectively (Figure 4). We observed that two upregulated, and two downregulated metabolic pathways overlapped between Pi starvation and recovery stages (Figure 4). Downregulated amino acid metabolism, including lysine degradation and tryptophan metabolism, maybe related to a developmental switch to cope with stress and recovery (Batista-Silva et al., 2019). Upregulated nitrogen metabolism and phenylpropanoid biosynthesis contribute to provide basic nutrients and metabolism for plant development, and to copy biotic and abiotic stresses (Limami et al., 2014; Dong and Lin, 2021). These results indicated that barley root phosphorylation and the succinylation protein response to Pi deficiency and recovery were dynamic, with differences at the pathway level.

**Figure 9:**
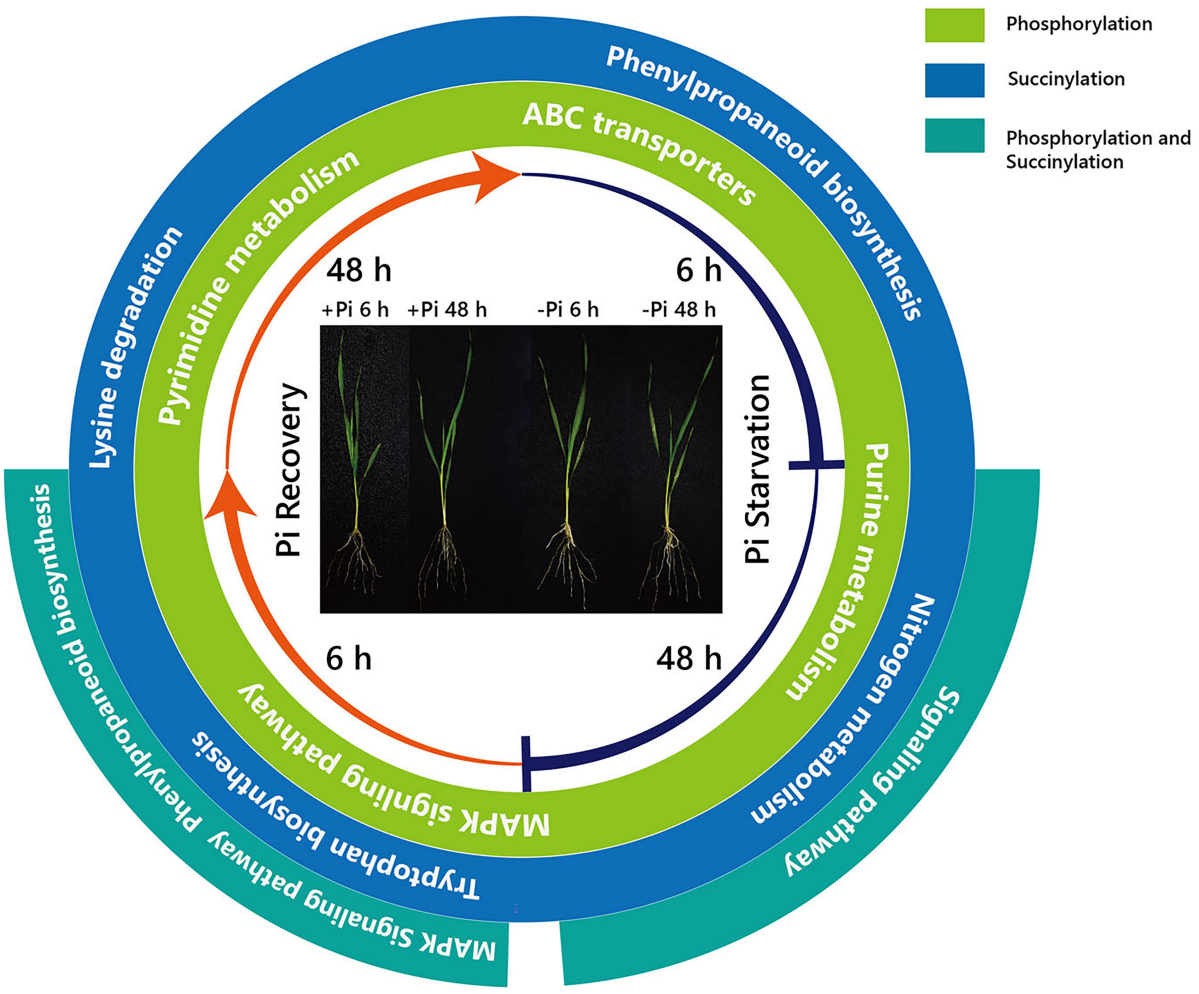
Summary of KEGG pathways of phosphorylated and succinylated proteins in response to Pi starvation and recovery. From the inside, the first and second circles show pathways overlapping the response of phosphorylation and succinylation proteins to Pi deficiency and recovery, respectively. The third circle shows pathways overlapping between the response of phosphorylation and succinylation proteins to deficiency and recovery. –Pi, Pi starvation; +Pi, Pi recovery. KEGG, Kyoto Encyclopedia of Genes and Genomes; Pi, intracellular phosphate.

Furthermore, surprisingly, the number of enriched metabolic pathways found to be overlapping the response of phosphorylation and succinylation proteins to Pi deficiency and recovery was very small. Only two pathways were identified: the MAPK signaling pathway, which was enriched after 48 h of Pi starvation, and the phenylpropanoid biosynthetic and MAPK signaling pathway, which was enriched after 6 h of Pi resupply (Figures 2–4). It is worth noting that among all 884 succinylated proteins, 275 were also phosphorylated, accounting for 31% of total succinylated proteins. Thus, we can speculate that MAPK signaling and phenylpropanoid biosynthesis play core roles in response to Pi stress. Consistent with previous transcriptome reports, phenylpropanoid metabolism was enriched in rice roots and shoots in short- and medium-term responses to Pi starvation and recovery (Secco et al., 2013). In this case, a total of eight core proteins belonged to the MAPK signaling pathway (Supplementary Table S15). Of these, the nucleoside diphosphate kinase family of proteins (NDPK; HORVU1Hr1G055440.1) was modified by both phosphorylation and succinylation at one site. The nucleoside diphosphate kinase family of proteins has been found to be involved in a wide range of biological processes including but not limited to signal transduction, and the response to salt stress (Luzarowski et al., 2017). Proteins modified only by phosphorylation include protein kinase superfamily proteins (HORVU4Hr1G001850.2 and HORVU2Hr1G075470.2; one site), respiratory burst oxidase homologue D (HORVU4Hr1G081670.1; one site), ethylene-insensitive protein 2 (HORVU5Hr1G050330.2; three sites), and respiratory burst oxidase homolog B (HORVU4Hr1G086500.9; three sites). In comparison, 40S ribosomal protein S6a (HORVU2Hr1G010870.8) was modified by only succinylation at five sites. Additionally, 15 core enrichment proteins belonging to the phenylpropanoid biosynthesis pathway were modified by phosphorylation and succinylation (Supplementary Table S15). Of these, four proteins were all identified as alcohol dehydrogenase, with from one to three phosphorylation sites. Alcohol dehydrogenase play a role in growth, development, and abiotic and biotic stresses in plants, such as cold stress regulation (Su et al., 2020), wounding (Kim et al., 2010), and lignin biosynthesis (Cheng et al., 2013). Similarly, only four proteins with succinylation one site were identified as peroxidase superfamily proteins, which are involved in plant development and the stress response (Bela et al., 2015). In addition, it is noteworthy that phenylalanine ammonia-lyase 2 (HORVU6Hr1G058820.1), which was related to the stress response with two succinylation sites, was enriched in core succinylation proteins. Finally, our global proteome analyses of phosphorylation and succinylation of barley root proteins covering the 48 h Pi starvation and 48 h Pi recovery stages, generated a comprehensive overview of the dynamic responses to Pi homeostasis for barley root proteins involved in different metabolic pathways (Figure 9) Phosphorylation and succinylation proteins related to MAPK signaling and phenylpropanoid biosynthetic pathways were relatively active in response to Pi stress.

## Conclusion

This study aimed to elucidate the underlying mechanisms of protein phosphorylation, and succinylation in response to Pi stress. Our data indicate that in a proportion of barley roots, phosphorylation and succinylation are dynamically regulated by Pi starvation and recovery treatments, which may be important for plants to cope with Pi stress conditions. Marked differences exist between phosphorylation and succinylation proteins in significantly enriched metabolic pathways during Pi starvation and recovery at the same time point. Furthermore, overlapping proteins modified by both phosphorylation and succinylation were primarily enriched in MAPK signaling, and phenylpropanoid biosynthetic pathways. Protein–protein interaction network analyses indicated that the response of central metabolic pathways to Pi starvation and recovery was significantly modulated by phosphorylation or, succinylation, or both. Our study provides new evidence for protein phosphorylation and succinylation regulating the activities of key proteins involved in plant responses to Pi starvation and recovery.

## Data availability statement

The mass spectrometry data from the succinylome and proteome have been deposited in ProteomeXchange with the dataset identifiers, PXD022052 and PXD022053, respectively.

## Author contributions

JW, CL, and PR carried out the proteomic analysis and drafted the manuscript. ZM, LY, BL, YM, and XM participated in material culture and performed the statistical analysis. HW and XS conceived of the study, and participated in its design. HW, ES, and KY helped to draft the manuscript. All authors have read and approved the final manuscript.

## Funding

This work was supported by the China Agriculture Research System (Grant CARS-05-04B-2); Industrial Support Project of Colleges and Universities in Gansu Province (2021CYZC-12); National Natural Science Foundation of China (31960426, 32160460, 32160496); Fuxi Talent Project of Gansu Agricultural University (Ganfx-03Y06); Key Projects of Natural Science Foundation of Gansu Province (20JR10RA507; 21JR7RA801).

## Conflict of interest

The authors declare that the research was conducted in the absence of any commercial or financial relationships that could be construed as a potential conflict of interest.

## Publisher’s note

All claims expressed in this article are solely those of the authors and do not necessarily represent those of their affiliated organizations, or those of the publisher, the editors and the reviewers. Any product that may be evaluated in this article, or claim that may be made by its manufacturer, is not guaranteed or endorsed by the publisher.

## Footnotes

^1^https://webblast.ipkgatersleben.de/barley_ibsc/downloads (Accessed July 30, 2022).

^2^http://www.genscript.com/psort/wolf_psort.html (Accessed July 30, 2022).
