# Supplementary material for: Global proteome analyses of phosphorylation and succinylation of barley root proteins in response to phosphate starvation and recovery
Source: Front Plant Sci. 2022 Aug 18;13:917652. doi: 10.3389/fpls.2022.917652 (PMC9433975; doi:10.3389/fpls.2022.917652)
Supplement: Supplementary file 1 [file Data_Sheet_1.ZIP › Additional files/Table S15.docx]

**Table S15. Related proteins belong to MAPK signaling pathway and phenylpropanoid biosynthesis were**

**modified both phosphorylated and succinylated.**

| **MAPK signaling pathway** | | | | | | | | | | | | | |
| --- | --- | --- | --- | --- | --- | --- | --- | --- | --- | --- | --- | --- | --- |
| Phosphoproteome | | | | | | | | **Succinylome** | | | | | |
| **ID** | | **Name** | | | | **PTM modified**  **Site** | | **ID** | | **Name** | **PTM modified**  **Site** | | |
| **HORVU1Hr1G055440.1** | | **Nucleoside diphosphate kinase family protein** | | | | **HORVU1Hr1G055440.1_118** | | **HORVU1Hr1G055440.1** | | **Nucleoside diphosphate kinase family protein** | **HORVU1Hr1G055440.1_87** | | |
| HORVU4Hr1G001850.2 | | Protein kinase superfamily protein | | | | HORVU4Hr1G001850.2_465 | | HORVU2Hr1G010870.8 | | 40S ribosomal protein S6a | HORVU2Hr1G010870.8_248  HORVU2Hr1G010870.8_271  HORVU2Hr1G010870.8_56  HORVU2Hr1G010870.8_47  HORVU2Hr1G010870.8_151 | | |
| HORVU5Hr1G050330.2 | | Ethylene-insensitive protein 2 | | | | HORVU5Hr1G050330.2_593  HORVU5Hr1G050330.2_462  HORVU5Hr1G050330.2_633 | |  | |  |  | | |
| HORVU4Hr1G086500.9 | | Respiratory burst oxidase homolog B | | | | HORVU4Hr1G086500.9_28  HORVU4Hr1G086500.9_81  HORVU4Hr1G086500.9_622 | |  | |  |  | | |
| HORVU2Hr1G075470.2 | | Protein kinase superfamily protein | | | | HORVU2Hr1G075470.2_154 | |  | |  |  | | |
| HORVU4Hr1G081670.1 | | Respiratory burst oxidase homologue D | | | | HORVU4Hr1G081670.1_106 | |  | |  |  | | |
| **Phenylpropanoid biosynthesis pathway** | | | | | | | | | | | | | |
| Phosphoproteome | | | | | | | **Succinylome** | | | | | | |
| **ID** | | | **Name** | | **PTM modified**  **Site** | | **ID** | | **Name** | | | **PTM modified**  **Site** | |
| HORVU5Hr1G062030.9 | | | Alcohol dehydrogenase | | HORVU5Hr1G062030.9_54  HORVU5Hr1G062030.9_52  HORVU5Hr1G062030.9_54 | | HORVU7Hr1G083550.1 | | Peroxidase superfamily protein | | | HORVU7Hr1G083550.1_240 |  |
| HORVU6Hr1G035420.6 | | | Cinnamyl alcohol dehydrogenase 5 | | HORVU6Hr1G035420.6_24 | | HORVU6Hr1G058820.1 | | Phenylalanine ammonia-lyase 2 | | | HORVU6Hr1G058820.1_236  HORVU6Hr1G058820.1_332 |  |
| HORVU4Hr1G087630.1 | | | Cytochrome P450 superfamily protein | | HORVU4Hr1G087630.1_134 | | HORVU1Hr1G023750.1 | | Peroxidase superfamily protein | | | HORVU1Hr1G023750.1_222 |  |
| HORVU0Hr1G016330.1 | | | phenylalanine ammonia-lyase 2 | | HORVU0Hr1G016330.1_583 | | HORVU2Hr1G125110.6 | | Peroxidase superfamily protein | | | HORVU2Hr1G125110.6_112 |  |
| HORVU7Hr1G118090.4 | | | HXXXD-type acyl-transferase family protein | | HORVU7Hr1G118090.4_41 | | HORVU1Hr1G066600.1 | | Peroxidase superfamily protein | | | HORVU1Hr1G066600.1_86 |  |
| HORVU3Hr1G089520.5 | | | beta glucosidase 42 | | HORVU3Hr1G089520.5_241 | |  | |  | | |  |  |
| HORVU7Hr1G030670.1 | | | hydroxycinnamoyl-CoA shikimate/quinate hydroxycinnamoyl transferase | | HORVU7Hr1G030670.1_225 | |  | |  | | |  |  |
| HORVU5Hr1G062030.9 | | | Alcohol dehydrogenase | | HORVU5Hr1G062030.9_52  HORVU5Hr1G062030.9_54 | |  | |  | | |  |  |
| HORVU0Hr1G012940.6 | | | Alcohol dehydrogenase | | HORVU0Hr1G012940.6_210  HORVU0Hr1G012940.6_20  HORVU0Hr1G012940.6_23 | |  | |  | | |  |  |
| HORVU3Hr1G097140.5 | | | Alcohol dehydrogenase | | HORVU3Hr1G097140.5_25 | |  | |  | | |  |  |
